# Concordance Between MR Safety Guidance Documents on Conditions for a Safe MR Environment

**DOI:** 10.1002/jmri.70444

**Published:** 2026-07-08

**Authors:** Ives R. Levesque, Véronique Fortier, Jorge Campos Pazmiño, Evan McNabb

**Affiliations:** ^1^ Medical Physics Unit McGill University Montréal Québec Canada; ^2^ Research Institute of the McGill University Centre Montréal Québec Canada; ^3^ Medical Imaging McGill University Health Centre Montréal Québec Canada; ^4^ Department of Medical Physics McGill University Health Centre Montréal Québec Canada

**Keywords:** facility design, hazard controls, MR environment, MR safety, MR safety guidance

## Abstract

**Background:**

Environmental conditions established at facility design, construction, updates, and/or procurement of specialized equipment are the foundation of safe MR practice. Establishing an institutional MR safety program must take into consideration the conditions of the MR environment, and staff training should be tailored to its features.

**Purpose/Hypothesis:**

To identify the concordance on conditions for a safe MR environment among a variety of widely known national guidance documents.

**Study Type:**

Analysis of guidance documents.

**Population/Subjects/Phantom/Specimen/Animal Model:**

Not applicable.

**Field Strength/Sequence:**

Not specific to field strength.

**Assessment:**

Information was extracted from national MR safety guidance documents about the expected conditions in a safe MR environment, and categorized among (1) facility design, (2) MR equipment selection, and (3) peripheral equipment. Commonalities and differences were identified.

**Statistical Tests:**

Descriptive statistics.

**Results:**

Six publicly available national guidance documents for MR safety were obtained, from countries with relatively high density of MR systems (between 9 and 38 MR systems per million inhabitants): Australia, New Zealand, Sweden, Switzerland, UK, and USA. Full or partial concordance was observed among many measures related to facility design (21/25 conditions), MR equipment features (7/9 conditions), and peripheral equipment (10/13 conditions).

**Data Conclusion:**

This work identified the common and divergent recommendations on facilities and equipment from a variety of guidance documents from national sources. Highlighting common (and uncommon) features recommended by guidance can help users identify weaknesses in their MR environment. This could inform jurisdictions/countries without official guidance and serve in the development of future guidance documents or MR safety training materials.

**Evidence Level:**

5.

**Technical Efficacy:**

1.

## Plain Language Summary

Human magnetic resonance imaging presents hazards to both patients and staff due to the use of magnetic fields to image the human body. The risks associated with these hazards are reduced through careful device engineering, facility design, staff expertise, institutional policies, and clinical procedures. Healthcare institutions rely on expert guidance to inform the necessary safety measures. In this work, six recognized national‐level guidance documents were analyzed to extract and compare the information related to facility design and equipment selection that establish the environment for safe practice of in vivo magnetic resonance.

## 1 Introduction

The medical community recognizes the risks involved with the practice of in vivo magnetic resonance (MR) imaging and spectroscopy. Clinical MR safety guidance exists to inform how clinical practitioners recognize and address the hazards present in the MR environment. Guidance focuses on three areas of concern: (i) safety of the MR system itself, through system design and engineering, (ii) bioeffects, addressed through a combination of system design standards and operational MR safety guidance, and (iii) safety of peripheral, wearable, or implanted devices within the MR environment, again through design, testing and clinical guidance. At the heart of MR safety practice lies the objective to mitigate risk for patients, healthcare staff, technical personnel, and/or researchers.

MR system safety has long been a topic of interest for the International Electrotechnical Commission (IEC), which has published and updated medical device safety standards applying to clinical MR systems since 1995, and as recently as 2022 [1]. While this international technical standard for human MR instrument safety recognizes that there are inherent hazards in the MR environment, it leaves the responsibility for risk mitigation to MR site operators. Clinical MR safety guidance is most commonly articulated in documents written by committees of experts on behalf of national organizations [2, 3, 4, 5, 6, 7]. The guidance is largely focused on policy, procedure, staff training, and expertise to mitigate risk. Research has documented needs for formal MR safety policy and practice [8, 9, 10, 11] and for MR‐Conditional equipment and emergency preparedness [12].

MR safety guidance documents are based on certain assumptions about the design and setup of the MR environment. These assumptions often rely on facility design, construction, and equipment selection to follow a set of industry or institutional guidelines [13]. For example, it might be assumed that the MR facility has specific layout features, or that certain peripheral equipment is present in the suite. As a result, guidance proposed in one jurisdiction may not carry over easily to another, depending on assumptions about facility design.

Scientific literature on the topic of facility design for MR safety emerged early in the history of clinical MRI, around 4 decades ago [14, 15, 16]: the hazard of ferromagnetic interactions and forces was perhaps the most prominent focus at the time. Notably, AAPM Task Group 20 [14] was formed to gather information and recommend paths for further research on MR site design. At least one healthcare organization has published a design guide [13]. The literature subsequently expanded to include more information on site design [17, 18], covering technical specifications and their impact on efficiency [17] and on vibrational and sound isolation [19]. More recent site design literature has considered intraoperative facilities [20].

In parallel, early publications in MR safety practice addressed exposure to magnetic fields [21, 22, 23], spawned the development of formal device safety guidance [24], which led to developments in clinical MR safety guidance [25, 26, 27, 28, 29] and occupational safety [30].

Consideration of site design has continued to progress in the past decade, building on the concept of safety by design [31, 32, 33]. Facility design for interventional suites captured attention during this period [31]. Recognizing that inadequate site design is an important contributing factor to MR safety failures leading to unsafe operations despite the best of operator knowledge, the concept of “site‐design‐as‐safety” has been proposed [33]. Indeed, facility design is a fundamental characteristic of safe operation, which can be classified as an “engineering control” in the hierarchy of hazard controls [34]. These considerations of site design developed alongside further advances in clinical MR safety guidance [35, 36, 37]. On the topic of safety equipment selection, ferromagnetic detectors have received attention in the research, suggesting that these should be used as complements to a robust safety program [38, 39].

Further to safe design and standards for MR system safety features, ergonomic issues followed in subsequent literature, notably resulting in time savings [40]. Perhaps more relevant to this work was the observation that reliance on staff to ensure safe practice was fragile and likely to suffer from design‐induced risk [41], urging the community to strengthen the human factors and ergonomics (HFE) elements in facility design and equipment procurement, thereby reducing the practice compliance component of MR safety.

Despite low reported MRI incident rates, documented events and evidence of under‐reporting highlight the limits of relying solely on operator competence. Studies show increasing rates of use of MRI [42] and increasing numbers of MR systems per capita [43]. Rates of incidents remain low, lying well below 1% of exams in studies spanning a range of institutions [44, 45, 46, 47]. Other studies cite RF‐field induced burns as the most reported patient injury [48, 49]. Projectile incidents accounted for 9% of events in one review [49] and added up to approximately 19 such incidents per year in another review [50]. These are encouraging numbers; however, there is cause for concern. Two studies observed severe under‐reporting of MR incidents [8, 10], a third concluded a common false sense of security in MR [11], and six fatal incidents from mis‐use of implantable infusion pumps were reported to the FDA over 2013–2019 [51].

The findings of reports on site design have continued to advance. One report suggested a formal categorization of MR safety practices into three areas—clinical (administrative), operational, and physical environment [52]—thereby treating the MR environment as a key ingredient of the MR safety framework. More recent work has examined MR safety as a design element among others in enhancing workflows and value in clinical MRI [53]. The drive to move towards workflows rather than checklists relies in part on structural environment features.

While much of the literature discussed above has found its way into the most recent guidance on clinical MR safety, most jurisdictions may not have locally enforceable MR safety guidance for clinical MR safety and, in addition, it is unclear whether the existing guidance acknowledges and includes MR‐safety‐oriented site design features. A critical assessment of the state of affairs is therefore well motivated.

The purpose of this work was to provide a comparative analysis of recommended conditions for the MR environment present in clinical MR safety guidance documents. Information about the conditions prescribed by various MR safety guidance documents was collected and compared, and concordance was assessed. Attention was focused on conditions related to facility, MR system, and peripheral equipment, all of which are typically selected prior to establishing safety policies and procedures.

## 2 Methods

Publicly and freely available MR safety guidance documents were collected and critically reviewed to compare the environmental conditions for facilities and equipment required and/or recommended in the guidance. Web searches were performed using the keywords “MR safety” and “guidance” paired with country names, proceeding in order of per capita density of MR systems, and adding other countries in and ad hoc manner to boost geographic representation. Data about numbers of MRI systems (total and per capita) [43] and country population [54] were retrieved to provide context about the representativity of the analysis. Documents were included in the analysis if (i) they aimed to provide broad and detailed guidance on MR safety policy and practice covering patients and personnel, (ii) they were published by recognized national‐level organizations, and (iii) contained a minimum amount of information pertaining to facility design features and equipment selection criteria. Documents such as proposals, surveys, opinions, and research articles that did not fit the above description were excluded. Guidance documents where the scope was deemed too narrow—e.g., aimed only at occupational exposure to magnetic fields—were excluded. The search workflow is illustrated in Figure 1.

**FIGURE 1:**
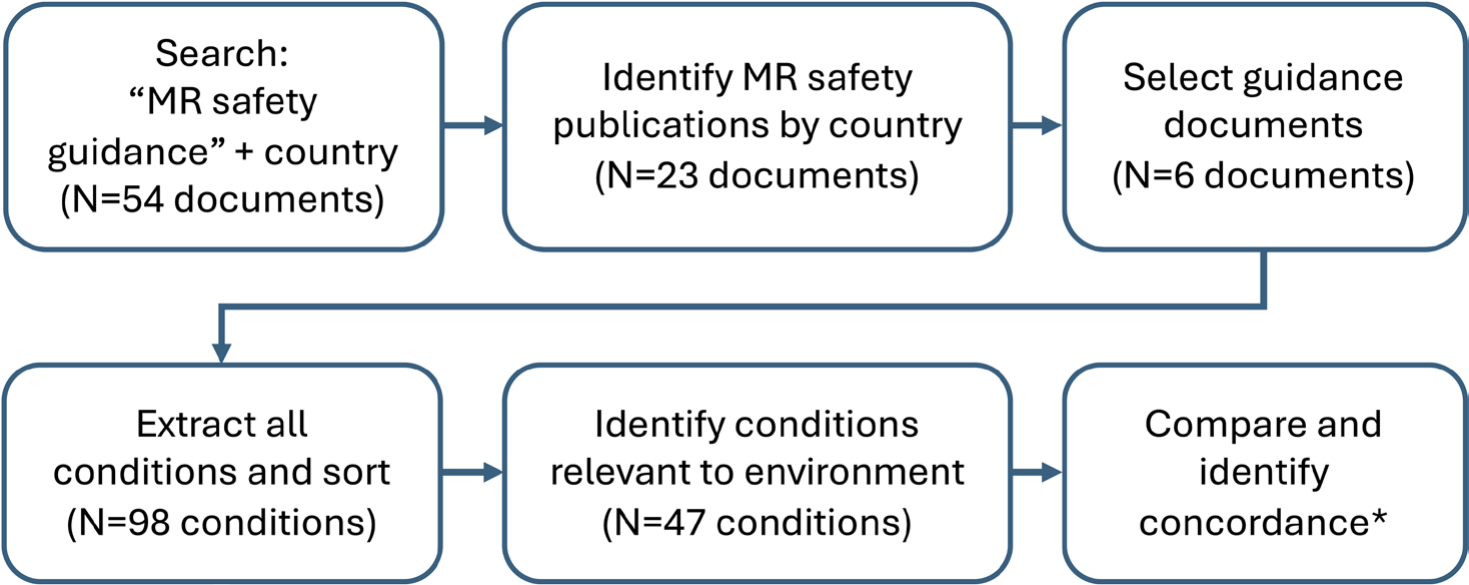
Workflow for literature search, selection of relevant documents, analysis of MR environment conditions, and identification of concordance. *Concordance is defined based on appearing in the guidance, as explained in the text.

Tables were constructed to collect the assumptions and environmental conditions needed to enable safe MR practice. The conditions were compared across available guidance documents. The concordance on environmental conditions was assessed to identify the most common requirements and was scored based on the number of documents where it appeared. Concordance between documents required mention in at least four of the six documents or a score of 4/6 = 67% or greater. This range was selected to align with the working definition of “consensus” commonly found in Delphi processes [55], quantified at 70%–80% agreement. Lower scores were described as “partial concordance,” defined as appearing in two or three of the six documents. True discrepancies were labeled as “no concordance,” appearing in one document at most.

Conditions were assigned to one of three categories: facility design, MR system, and peripheral equipment. Concordance on each condition within every category was scored by tabulating the number of conditions appearing as “concordance,” “partial concordance,” and “no concordance.” To assess the potential deviations of the guidance documents against the others, each document was assigned a score based on the inclusion of each environment condition (out of the total number of conditions). Then, the deviation of each document relative to the median concordance score was calculated.

## 3 Results

Among 54 countries included in the search for this work, documents pertaining to MR safety were identified originating from 23 countries. Many of the documents were scientific publications of operator or site surveys, or very brief guidance documents. Among these 23 documents, six nationally recognized guidance documents generated by professional organizations representing six countries were identified and selected for analysis (Figure 2). These guidance documents have all been published since 2018 and are representative of the most recent recommendations. Previous versions of these documents were not analyzed. Figure 3 provides a summary of the timeline of publication of 25 guidance documents in the MR safety literature over the past 23 years.

**FIGURE 2:**
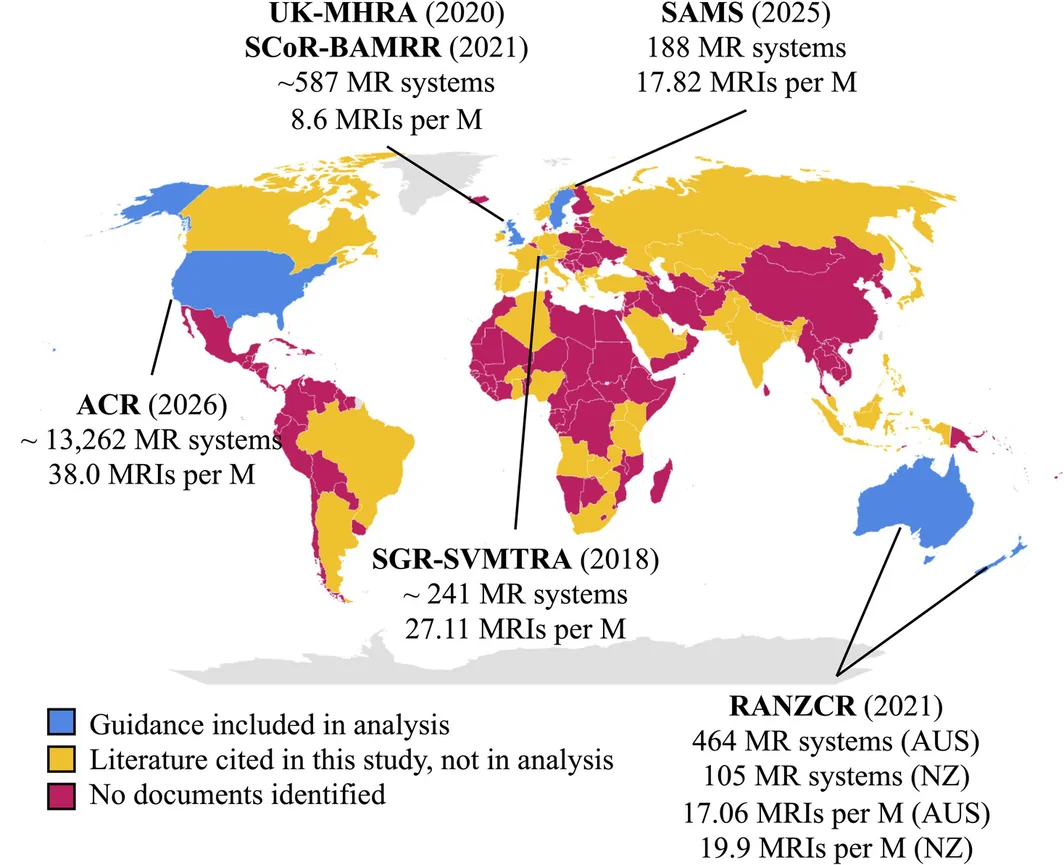
Visual summary of countries where MR safety guidance literature was identified and included in this study. Blue = included in the study (*N* = 6); yellow = MR safety literature identified but not directly relevant to, nor included in, the study; red = no literature identified. “per M” = per million inhabitants.

**FIGURE 3:**
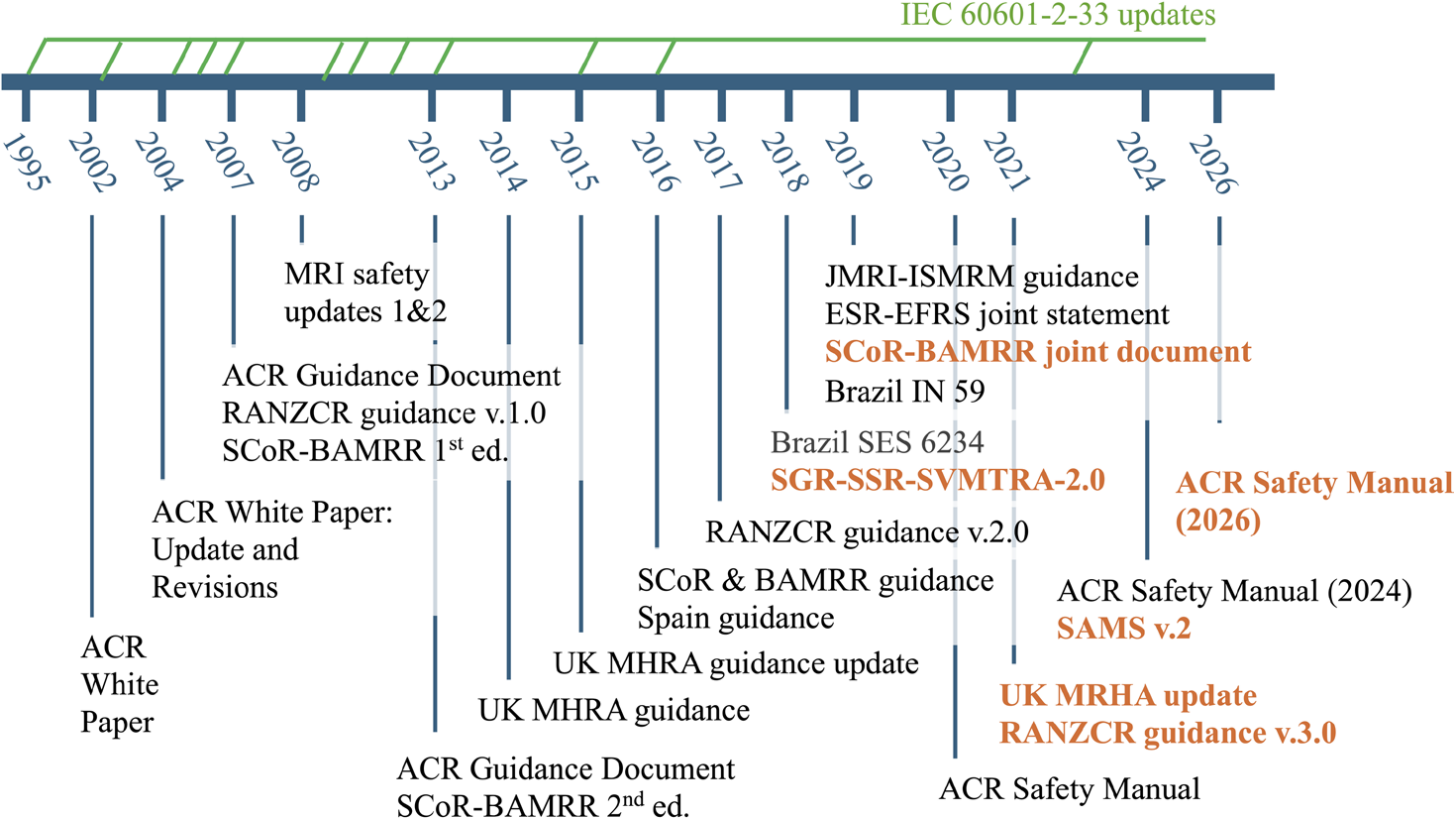
Timeline of publication of MR safety guidance identified in this work. The six documents retained for analysis are highlighted in orange.

These six publicly‐available national guidance documents for MR safety [2, 3, 4, 5, 6, 7] were analyzed, originating from countries with a relatively high density of MR systems (between 9 and 38 MR systems per million inhabitants). Demographic data of the countries of origin of the MR safety guidance are reported in Table 1.

**TABLE 1:** List of safety documents with countries of origin and demographic data.

Title	Source^a^	Year	Country	Population [54]	MR per M inhabitants [43]	Estimated MR systems
“Empfehlung zur Sicherheit von Magnetresonanz‐Untersuchungen am Menschen,” version 2.0 [5]^b^	SGR‐SVMTRA	2018	Switzerland	9 M	27.11	241
“Safety in MRI” [4]	SCoR/BAMRR	2020	UK	69.6 M	8.6	587
“Safety Guidelines for Magnetic Resonance Imaging Equipment in Clinical Use” [2]	UK MHRA	2021	UK	*idem*	*idem*	*idem*
“MRI Safety Guidelines Version 3.0” [3]	RANZCR	2021	Australia New Zealand	27 M 5.3 M	17.06 19.9	464 105
“Swedish National Recommendations for MR Safety 2025” [6]	SAMS	2025	Sweden	10.7 M	17.82	188
“ACR Manual on MR Safety” [7]	ACR	2026	USA	349 M	38.0^c^	~13,262^c^
			Totals:	~469 M	—	~14,850

*Note:* Data are ordered by year of publication of the guidance document, from oldest to most recent.

Abbreviations: ACR = American College of Radiology; ACR Committee on MR Safety; RANZCR = Royal Australian and New Zealand College of Radiology; SAMS = *Svenska Alliansen för Magnetkamera Säkerhet*, Swedish Alliance for MR Safety; SCoR/BAMRR = Society and College of Radiographers/British Association of MR Radiographers; UK MHRA = United Kingdom Medicines and Healthcare products Regulatory Agency.

^a^ SGR‐SVMTRA = *Schweizerische Gesellschaft für Radiologie*—*Schweizerische Vereinigung der Radiologiefachpersonen*.

^b^ Documents published in languages other than English were machine‐translated (translate.google.ca).

^c^ Data for USA is from 2021; total MR systems estimated by multiplying by 2026 population.

In total, 98 MR safety conditions were identified: of these, 47 were identified as environment conditions needed to enable safe MR practice. Each condition was assigned to one of three categories: facility design (*N* = 25), MR system (*N* = 9), and peripheral equipment (*N* = 13). These are listed in Tables 2, 3, 4, respectively. Each table indicates whether the condition appears in each of the 6 guidance documents and labels the level of concordance.

**TABLE 2:** Facility‐related environment conditions (*N* = 25).

Description	ACR	SAMS	RANZCR	UK MHRA	SCoR/BAMRR	SGR‐SVMTRA	Concordance?
Access to MR suite is controlled by locks	Y	Y	Y	Y	Y	Y	Yes
Signage indicating that magnet is always on/magnet hazards	Y	Y	Y	Y	Y	Y	Yes
Cryogen vent location is known, secured, and accessible to workers	Y	Y	Y	Y	Y	Y	Yes
0.9 mT line is marked; other field contour lines marked as needed	Y	Y^c^	Y	Y	Y	—	Yes
Helium exhaust fan is installed and functional	Y	Y	Y	Y	Y	—	Yes
Document provides a summary of facility design features	Y	Y	Y	Y	Y	—	Yes
Changing & patient preparation area is available, distinct from MRI room	Y	Y	Y	Y	Y	—	Yes
Designated resuscitation area located outside 0.5/0.9 mT zone	Y	Y	Y	Y	Y	—	Yes
Projectile zone marking at specified field contour line	Y^a^	Y	—	Y^b^	Y^b^	Y	Yes
Oxygen monitor alarm is installed	—	Opt	Y	Y	Y	—	Yes
Facility layout is amenable to safety zone system	Y	Y	Y	Y	—	—	Yes
Secure storage for belongings	Y	Y	Y	Y	—	—	Yes
Passive pressure release system	Y	—	Y	Y	—	Y	Yes
Operator has line of sight view to Zone IV	Y	Y	Y	Y	—	—	Yes
Available area for sedation outside Zone IV	Y	—	Y	Y	Y	—	Yes
Direct supply of gases available in MRI suite	Y	—	Y	Y	—	—	Partial
Designated cryogen storage area	—	—	Y	Y	—	—	Partial
Alarms (beyond squeeze ball), e.g., for emergencies	—	—	Y	Y	—	—	Partial
Tether points are installed	Y	Y	—	—	—	—	Partial
Designated zone for full stop/final check	Y	Y	—	—	—	—	Partial
Designated area for patient transfer and ferromagnetic quarantine	Y	—	Y	—	—	—	Partial
Stable power supply with temporary back up as needed	Y	—	—	—	—	—	No
Guidance provided for many magnets sharing Zone III	Y	—	—	—	—	—	No
Designated post‐screening patient holding zone	Y	—	—	—	—	—	No
Storage for transport and maintenance equipment	Y	—	—	—	—	—	No

*Note:* A “Y” indicates that the condition is mentioned in the guidance document, while a “—” indicates that it is not.

Abbreviations: “—” = not mentioned; NR = not recommended; Opt = optional.

^a^ Projectile area mentioned without a field value.

^b^ Field contour values vary.

^c^ Sweden has kept 0.5 mT as the exclusion field value [6].

**TABLE 3:** MR system‐related environment conditions (*N* = 9).

Description	ACR	SAMS	RANZCR	UK MHRA	SCoR/BAMRR	SGR‐SVMTRA	Concordance?
MR system conforms to IEC Standard 60601‐2‐33	Y	Y	Y	Y	Y	Y	Yes
Emergency stop, emergency power off, and quench buttons are all accessible	Y	—	Y	Y	Y	Y	Yes
Squeeze ball is available for patient	Y	—	Y	Y	—	Y	Yes
MR system and/or facility has airflow for temperature management	—	Y	—	Y	Y	Y	Yes
Patient table is free to move during power failure	Y	—	Y	Y	—	—	Partial
Patient intercom available	Y	—	—	Y	—	—	Partial
Local transmit coil is available	Y	—	—	Y	—	—	Partial
MR system enables helium check by user	—	—	—	—	Y	—	No
MR system software assistance in meeting implant conditions	—	—	—	—	Opt	—	No

*Note:* A “Y” indicates that the condition is mentioned in the guidance document, while a “—” indicates that it is not.

Abbreviations: “—” = not mentioned; NR = not recommended; Opt = optional.

**TABLE 4:** Equipment‐related environment conditions (*N* = 13).

Description	ACR	SAMS	RANZCR	SCoR/BAMRR	UK MHRA	SGR‐SVMTRA	Concordance?
MR conditional fire extinguisher	Y	Y	Y	Y	Y	Y	Yes
MR conditional equipment for hybrid and/or interventional	Y	Y	Y	Y	Y	—	Yes
Available monitoring equipment is MR Conditional	Y	Y	Y	Y	Y	Y	Yes
Ancillary equipment in MRI suite is all MR Safe or Conditional	Y	Y	Y	Y	Y	Y	Yes
Hand‐held test magnet available	Y	Y	Y	—	Y	Y	Yes^a^
Life support, AED	Y	Y	Y	Y	Y	—	Yes
Temperature and humidity monitors	—	Y	Y	Y	Y	Y	Yes
Availability of MR conditional stretcher	Y	Y	Y	—	Y	—	Yes
Adapted tools for maintenance/cleaning	Y	Y	Y	—	—	Y	Yes
Phantom storage, handling, and emergency procedures	Y	—	—	Y	Y	—	Partial
Ferromagnetic metal detectors	Opt	Opt	Opt	—	Opt	—	No
Metal detectors	NR	—	Y	—	NR	—	No
Inventory of ancillary equipment	—	—	—	Y	—	—	No

*Note:* A “Y” indicates that the condition is mentioned in the guidance document, while a “—” indicates that it is not.

Abbreviations: “—” = not mentioned; NR = not recommended; Opt = optional.

^a^ Recommended test magnet strength varies or is not specified.

Overall, this analysis shows that concordance is common among many measures related to facility design (full or partial concordance on 15 + 6 = 21 conditions), MR system features (full or partial concordance on 3 + 4 = 7 conditions), and peripheral equipment (full or partial concordance on 9 + 1 = conditions). Full concordance over MR environment conditions was most common (28 conditions across all categories). Absence of concordance was noted for 9 conditions, distributed across the 3 categories. These numbers are plotted in Figure 4.

**FIGURE 4:**
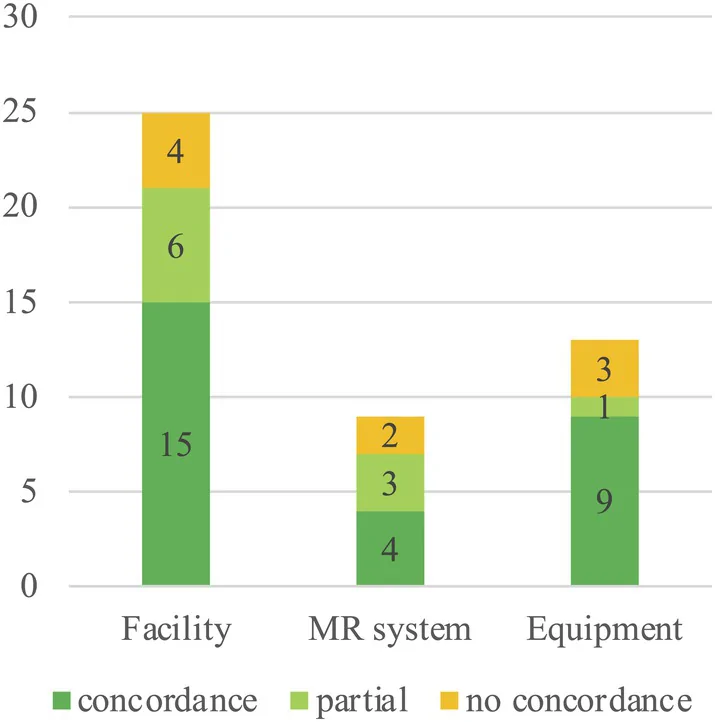
Summary observations of concordance for MR equipment and environment conditions among six MR safety guidance documents from national organizations. Full, partial, and absence of concordance are defined in the text. A total of 47 conditions were identified, with over half related to facility design conditions.

The guidance documents varied in terms of overall concordance with each other, as reflected in the deviations from the median concordance score (Figure 5). The median concordance score was 28.5, and deviations from this ranged from −12.5 to +9.5, with most within the ±5 range. In this analysis, a positive score indicated that a document mentions more of the MR environment conditions than the median among all the documents. One document (SGR‐SSR‐SVMTRA) showed greater negative deviations from the other scores. There appears to be a trend towards high positive scores in more recent documents, although the SAMS document breaks with this trend.

**FIGURE 5:**
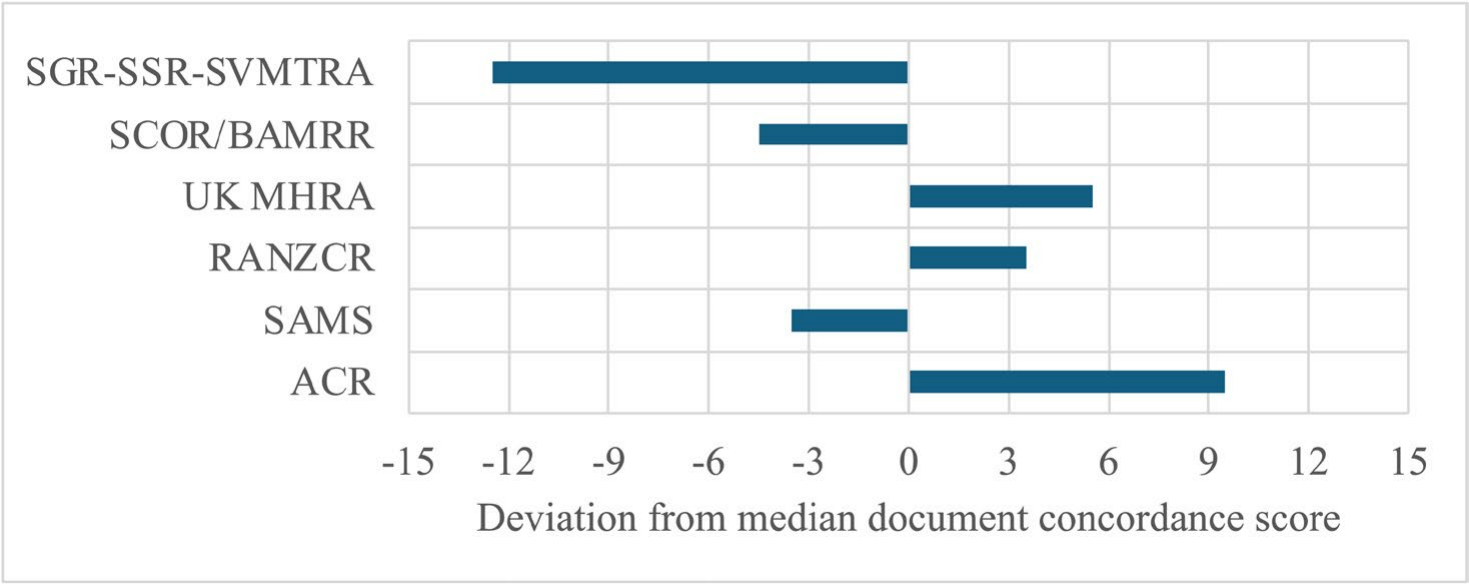
Concordance varies among guidance documents, as reflected in this plot of the deviation of the concordance score for each document. First, a document score calculated on whether each condition is mentioned in the document (out of a possible 47) is provided. Then the deviation from the median (= 28) is calculated and plotted. A score of zero on this scale indicates that a document mentions the median number of conditions among the guidance documents considered in this study. Documents are ordered by year of publication from oldest (top) to most recent (bottom).

## 4 Discussion

MR safety practice has progressed considerably over the decades since the first clinical experiences, alongside growing use of MRI in the fields of medicine and research. MR safety guidance, reviewed in this work, has been developed to inform and instruct professionals and steer them towards best practices. As discussed, considerations for the MR working environment are too often an afterthought and should be reframed as an integral part of MR safety [33].

This work sought to identify concordance conditions for the MR work environment, including facility design, MR system, and peripheral equipment, among six widely available clinical MR safety guidance documents. A concordance scale was designed based on accepted criteria for identifying consensus, with score ranges attributed the labels of “concordance,” “partial concordance,” and “no concordance.”

This systematic study identified 47 distinct conditions that should feature in a modern MR facility, and organized them into three overarching categories, providing a structured framework for understanding their relevance within the broader landscape. By assessing whether these conditions appear within six nationally recognized guidance documents [2, 3, 4, 5, 6, 7], areas of alignment were identified that highlight emerging consensus within the field. Notably, the vast majority of conditions demonstrated at least partial agreement across sources, and a substantial proportion reached full concordance, underscoring widespread acceptability of recommendations despite variations in scope and methodology among the guidance documents. Together, these findings reinforce the value of synthesis across guidance documents in clarifying areas of concordance and informing more consistent clinical MR safety practice.

This comparative analysis of national safety guidance documents highlights the common conditions used to mitigate risk in a safe MR environment and underlines differences among guidance documents. These observations are particularly important to inform MR safety practice in jurisdictions where local guidance is absent, helping to answer questions about which guidance to follow and the desirable environment conditions to establish for safe MR practice. Conditions with high concordance are straightforward to recommend, while those achieving partial concordance could easily be addressed via adjustments to the literature. Conditions where concordance is not present identify potential areas where research is needed.

Among the guidance documents, the UK MHRA document [2], the ACR Manual on MR Safety [7], and the manual from the RANZCR [3] were observed to have the highest concordance, in that order. The ACR Manual notably features an entire appendix on facility safety design guidelines. Interestingly, 13 conditions did not feature in the ACR guidance despite mentions in other documents. Differences between guidance documents arose from specific conditions appearing in some documents but not in others. The concordance of guidance documents appears to be growing over time, as the more recent documents tended to receive a higher positive concordance score. It can be speculated that documents with lower concordance scores may have overlooked certain desirable MR environment conditions or taken them for granted with respect to the establishment of MR facilities.

Observations were made about specific conditions. Concordance was notably absent on the availability of tether points, on a designated zone for full‐stop‐and‐final‐check, and on the use of metal detectors. First, tether points (structural wall or floor anchors meant to secure portable equipment in Zone III or IV) are an uncommon yet useful facility design feature. These can be used to secure MR Unsafe equipment outside of the fringe field to avoid their inadvertent introduction, or within the MR environment to ensure that MR Conditional equipment is kept in a safe location. It would stand to reason that they would deserve mention in safety guidance. Second, the practice of full‐stop‐and‐final‐check is a commonly used tool in the healthcare field to coordinate teams ahead of potentially hazardous procedures (e.g., in surgery or interventional radiology), such that having a designated zone can serve as a reminder to staff to conduct this crucial step. Third, the use of conventional metal detectors is the only condition where there is not only absence of concordance, but true contradiction between guidance documents, and this would merit further attention from the MR safety community. In relation to this, while there has been increasing interest in ferromagnetic metal detectors [38, 39], these do not appear among concordant conditions.

There were variations among field values to be used to demarcate exclusion zones related to devices. While selected guidance (ACR) is aligned with the IEC move to a 0.9‐mT exclusion zone for active devices, another recent publication (SAMS) maintained the 0.5 mT specification to remain aligned with national regulation. Additionally, some publications (e.g., RANZCR) specify a field value to be marked as the projectile zone; other texts (e.g., ACR) specifically choose not to specify a field line to mark as the value will be dependent on the item, choosing instead to rely on the more conservative 0.9 mT exclusion zone for ferromagnetic devices.

By identifying the environmental conditions and design features recommended by national MR safety guidance documents, this work recognizes the importance of engineering controls in a hierarchical approach to hazard control [34]. Failure on the part of institutions to consider MR environment design features and equipment selection from the beginning will require the establishment of policies and procedures later to compensate for gaps in safety. From the perspective of the hierarchy of hazard controls, this means substituting a weaker “administrative” control (the second weakest level of control, which relies on decision making and compliance of personnel and/or patients) in the absence of a stronger control (such as an “engineering control” or a “substitution”) that can be designed, built, installed, or purchased. The weaker control will ultimately expose staff and patients to a greater risk.

Attempts at palliating gaps in safety design of MR facilities are most likely to be implemented as administrative controls, i.e., policy and procedures to be followed by staff. In the absence of facility safety features, the need arises for additional policy, procedures, and training. For instance, increased habits of vigilance and oversight of the facility will be required for deficiencies in facility design and access controls (e.g., facilty layout not amenable to zoning, or absence of operator line‐of‐sight to the MR system and access doors). A lack of secure storage for patient belongings may lead to patients not declaring personal items and bringing them into Zone IV, which imposes the burden of increased screening and vigilance. The absence of marking of various magnetic field exclusion zones might be compensated by the use of other physical landmarks (e.g., doorways) possibly leading to limitations on procedures and/or operational flexibility. Missing or non‐compliant peripheral equipment may lead to, at worst, unnecessary risk‐taking (such as risking MR Unsafe equipment in Zone IV, or forgoing patient monitoring), or become a limiting factor on available procedures. The absence of dedicated emergency life support equipment will restrict certain procedures, such as examination in the presence of cardiac implantable electronic devices or under anesthesia. Missing or lacking patient communication devices may impose that a staff member remain in the suite during active scanning. In some instances, such as missing emergency features (e.g., electrical power off control) or non‐IEC‐compliant MR systems, no amount of policy or procedures will fill the void. In other cases, such as the absence of additional ventilation for quench situations or of MR Conditional fire extinguishers, emergency policies and procedures will need to be adapted. Possible palliation measures to be used in the absence of specific conditions, would be an excellent topic for future consideration. Ultimately, these additional practice measures, when possible, will likely increase the burden on the attention of healthcare staff.

Reported incident data can be connected to facility design and/or choices in peripheral equipment. A 2019 investigation of MR adverse events over a 10‐year period in the USA—a leading market in the use of MR (38 MR systems per capita [43]) and a source of authoritative MR safety guidance [7]—revealed that the most common incidents were thermal (59%), mechanical (11%), and projectile (9%). Of the thermal events, for example, many involved contact with ECG leads (15%) or pulse oximeters (3%), both avoidable when the correct peripheral equipment is made available and used by attending staff. Most projectile incidents involved objects commonly encountered in the MR environment, such as transport and mobility devices, gas cylinders, patient monitoring equipment, carts, and IV poles, all of which can be sourced MR Conditional. In addition to sourcing MR Conditional equipment, the risk of projectile incidents can be reduced by access control (which depends on facility design) and potentially by ferromagnetic detectors (although this recommendation did not reach concordance).

This study has a few limitations that should be acknowledged. First, our choice to define concordance as a score of 4/6 documents was relatively permissive, and modifying the scale could influence interpretation. Second, some national guidance documents—such as those from Spain [56] and the Netherlands [57]—were not incorporated due to their narrow scope, which prioritizes issues related to worker safety while not fully addressing broader aspects such as patient safety or MR suite design. Third, certain relevant guidance could not be evaluated because it was not readily accessible. In Japan, guidance reportedly exists in two sources identified in the search. One is cited as reference 8 of this document [58]: efforts to obtain a copy of the cited 2014 document “MRI safety‐principles, standards and clinical concerns” from The Safety Committee of the Japanese Society for Magnetic Resonance in Medicine were unsuccessful. The other is the JSMRM website (machine‐translated to English), which linked to a two‐page document entitled “Guidelines for the Safe Operation of Clinical MRI (latest revision 2025, machine translated from Japanese). This document, while prescriptive in terms of MR safety practice, does not provide adequate details about the MR environment conditions to be included in this study. Finally, one document published exclusively in a language other than English was included in this work by using machine translation. Although this exercise enabled broader international representation, it also introduced the possibility of misinterpretation or loss of nuance, potentially affecting the accuracy of the synthesized findings.

Other notable absences from the list of guidance document include the following selection, where no guidance documents were identified for this analysis. In Brazil, two very brief guidance documents have previously been reviewed and compared by Clemente and Semmler [59]: one from the State of Minas Gerais [60] and a national document [61]; neither of these constituted a complete guidance document within the scope of the analysis. In Canada, the Canadian Association of Radiologists maintains practice guidelines in MRI, but not specific to MR safety, and recently retired its 2011 Standard on MRI, which had a one‐page section on MRI safety. The Canadian Association of Medical Radiation Technologists publishes practice guides for its members on its website. In India, a potential MRI Safety Board has been discussed [62], in parallel to results from a survey of radiographers [63]. Publications from China [64] and Russia [65] discussed their respective regulations and context, but fell outside the scope of this analysis.

A possible consequence of the increased risk from inadequate facility design or equipment selection for MR safety is the potential for greater cost (in capital, time, or human resources) to an institution in the long run due to a higher potential for incidents. While speculative, it is easy to imagine that saving on expenses at the design and construction stage may lead to greater costs due to safety risks and related incidents over the lifetime of the facility. The analysis of this trade‐off, considering the potential savings at facility construction versus costs incurred from incidents, could be a valuable research exercise.

## 5 Conclusion

Environmental conditions to be established at facility design, construction, or updates, and/or through procurement of specialized equipment are primordial in establishing the foundation for safe MR practice. This work summarized the recommendations on facilities and equipment from a variety of guidance documents from national sources. The analysis could serve in the development of best practices in the design of facilities and equipment selection for MR safety, in the development or revision of future guidance documents, and/or of MR safety training materials.

## Funding

This work was supported by the Montreal General Hospital Foundation, The Research Institute of the McGill University Health Centre, and the Fond de recherche du Québec—Santé (Chercheur Boursier).

## Conflicts of Interest

The authors declare no conflicts of interest.
